# A refined kidney tumor nephrometry system employed to screen pediatric patients who are eligible for nephron sparing surgery

**DOI:** 10.3389/fped.2024.1501560

**Published:** 2025-01-07

**Authors:** Mingchuan Huang, Yingchun Fei, Zhihai Zhong, Hong Jiang, Longshan Liu, Juncheng Liu, Huanxi Zhang, Jun Li, Zhe Xu, Pengfei Gao, Changxi Wang

**Affiliations:** ^1^ Department of Pediatric Surgery, The First Affiliated Hospital, Sun Yat-sen University, Guangzhou, China; ^2^ Organ Transplant Center, The First Affiliated Hospital, Sun Yat-sen University, Guangzhou, China; ^3^ Guangdong Provincial Key Laboratory of Organ Donation and Transplant Immunology, The First Affiliated Hospital, Sun Yat-sen University, Guangzhou, China; ^4^ Guangdong Provincial International Cooperation Base of Science and Technology, The First Affiliated Hospital, Sun Yat-sen University, Guangzhou, China

**Keywords:** thrombus, nephron sparing surgery, radical nephrectomy, pediatric, Wilms tumor

## Abstract

**Purpose:**

Comprehension of the anatomical characteristics of pediatric kidney tumors is crucial for making surgical decisions. Previous kidney tumor nephrometry systems failed to incorporate two significant factors: tumor thrombus and multifocality. We develop a refined nephrometry system based on a comprehensive understanding of the characteristics exhibited by pediatric kidney tumors.

**Methods:**

The TUMORS nephrometry scoring system comprises 6 indicators, including tumor (T)hrombus, (U)rinary collecting system involvement, (M)ultiple tumors, (O)utward property, (R)adius, and (S)ite relative to the polar lines. Each renal unit was assessed and scored independently. The complexity characteristics of kidney tumors were summarized, and the correlation was compared with RENAL nephrometry system. Furthermore, the complexity of kidney tumors was compared across different surgical procedures.

**Results:**

A total of 43 patients were enrolled, involving 70 kidney units. Radical nephrectomy (RN) was performed on 13 kidneys, while the remaining 57 kidneys underwent nephron sparing surgery. In the NSS group, tumors in 37 kidneys were resected *in vivo*, whereas 20 kidneys underwent tumor resection *ex vivo* followed by kidney autotransplantation. According to the TUMORS nephrometry scoring system, there were 13, 34 and 23 kidney units classified as low, moderate and high complexity, respectively. Tumors that underwent RN or *ex vivo* removal exhibited higher complexity. The complications and positive margins of NSS were not statistically significant in relation to tumor complexity.

**Conclusion:**

The TUMORS nephrometry scoring system holds significant guidance for the decision of surgical protocol and can be applied to the preoperative evaluation.

## Introduction

The field of kidney tumor anatomy has witnessed the emergence of various methodologies, including the RENAL, PADUA, and C-index systems (1–3). The aforementioned systems provide an objective description of tumor size, location, depth, and proximity to the renal sinus. As a result, they significantly contribute to guiding the feasibility of nephron sparing surgery (NSS) and evaluating postoperative complications (4). The validation of these methodologies, however, has primarily focused on adult renal cell carcinoma. Pediatric kidney tumors are often diagnosed when they have already reached a significant size, resulting in missed opportunities for implementing NSS.

Wilms tumor (WT) is the predominant type of pediatric kidney tumor, and the tumor thrombus, when present, invades the renal vein and extends into the inferior vena cava, accounting for approximately 4%–15% of WT patients; moreover, a smaller proportion of cases (approximately 0.7%–1%) exhibit extension into the right atrium (5–7). Approximately 5% of WT patients present with multifocal tumors (8). However, the existing systems fail to include these characteristics.

Tumor removal remains the mainstay for renal tumors. It should be noted that NSS is recommended for bilateral WT (BWT) and highly selective unilateral renal tumors in pediatric patients. The objective of this study is to develop a refined kidney tumor nephrometry system in pediatric patients, aiming to accurately delineate the anatomical characteristics and assess its potential utility for selecting candidates suitable for NSS.

## Methods

Based on the RENAL nephrometry scoring system (1), we proposed the TUMORS nephrometry system. The TUMORS nephrometry system comprises 6 descriptors, namely (T)hrombus, (U)rinary collecting system involvement, (M)ultifocality, (O)utward property, (R)adius, and (S)ite relative to the polar lines. The TUMORS nephrometry system had updated the RENAL nephrometry scoring system to include thrombus and multifocality. The first variable is (T)hrombus: when there is no tumor thrombus present in the renal vein and inferior vena cava, this variable receives a score of 1. Conversely, if a tumor thrombus is detected, the total score for the renal tumor unit is directly assigned as 16 points, without considering the status of the other five variables. The second variable is the (U)rinary collecting system: there is no invasion of the urinary collecting system by the tumor, scoring 1. If the tumor is in close proximity to the urinary collecting system, but there is no gross/microscopic hematuria, a score of 2 is assigned. A score of 3 is given when the tumor clearly invading the collecting system, or the tumor is in close proximity to the urinary collecting system with gross/microscopic hematuria. The third variable is (M)ultifocality: there is single tumor, which scored 1; the presence of two tumors result in a score of 2; and a score of 3 is assigned when three or more tumors are present. The remaining three variables are (O)utward property, (R)adius and (S)ite, which correspond to (E), (R) and (L) of the RENAL nephrometry system respectively (Table 1).

**Table 1:** TUMORS nephrometry scoring system.

	1 pt	2 pts	3 pts
(T)hrombus	Without tumor thrombus	a	
(U)rinary collecting system	Without invasion	Tumor is close to the urinary collecting system but without gross/microscopic hematuria	Tumor invasion, or tumor is close to the urinary collecting system with gross/microscopic hematuria
(M)ultifocality	single mass	2 masses	≥3 masses
(O)utward property	≥50%	>0% but <50%	0%
(R)adius (maximal diameter in cm)	≤4	>4 but <7	≥7
(S)ite relative to the polar lines	Entirely above the upper or below the lower polar lines	≤50% mass crosses polar line	≥50% mass crosses polar line; mass crosses axial renal midline; mass is entirely between the polar lines

^a^ The total score of the direct definition is 16 points if tumor thrombus is present.

The scores for the 6 descriptors were assigned respectively, as detailed in Table 1. If there were multiple masses within a single renal unit, (R) was scored according to the largest diameter observed, (U) was scored according to the mass closest to the urinary collecting system, while (S) was scored according on the mass nearest to the pole lines. Taking into consideration these six descriptors, the scoring for kidney tumors ranged from 6 to 16 points. These scores were further categorized into low complexity tumors (6–8 points), moderate complexity tumors (9–12 points), and high complexity tumors (13–16 points).

We retrospectively reviewed the surgical specimens and anatomical characteristics of 132 patients with renal tumors younger than 18 years of age who were admitted to the First Affiliated Hospital of Sun Yat-sen University from May 1, 2016 to December 1, 2023. These patients underwent contrast enhanced computed tomography before surgery. The study subjects underwent either unilateral or bilateral nephron sparing surgery (NSS). According to TUMORS nephrometry system, each renal unit was assessed independently. Continuous variables were given as mean ± standard deviations or median with ranges, and categorical variables were given as frequencies or proportions. Correlations were analyzed with Spearman correlation analysis. The Student's *t*-test, the Mann-Whitney U test, and Pearson *x*^2^ test were used, as appropriate. Statistical analysis was performed using SPSS version 26.0 software for Mac. For all statistical analyses, a two-sided *P* < 0.05 was considered statistically significant.

This study was approved by the Institutional Review Board of the First Affiliated Hospital of Sun Yat-sen University (IIT-2023-066). Due to its retrospective nature, the informed patient consent was waived.

## Results

**Figure 1:**
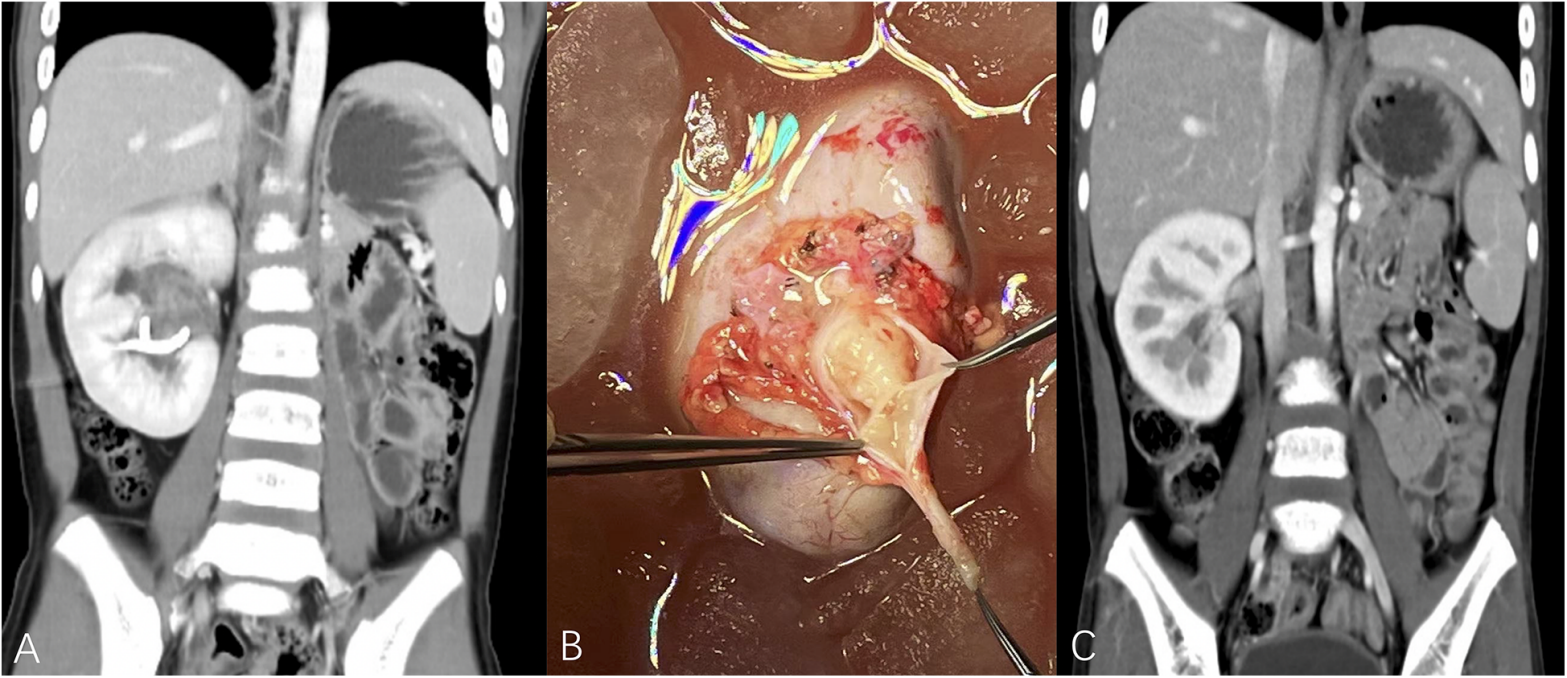
**(A)** Metachronous right kidney tumor after left RN, TUMORS – NS (right kidney unit) = 1 + 3 + 1 + 3 + 1 + 3 = 12. **(B)** Patient underwent tumor removal *ex vivo* with kidney auto-transplantation, polypoid tumor was visible in the urinary collecting system. **(C)** The imaging data of postoperative reexamination. (TUMORS – NS: TUMORS system - nephrometry score).

**Figure 2:**
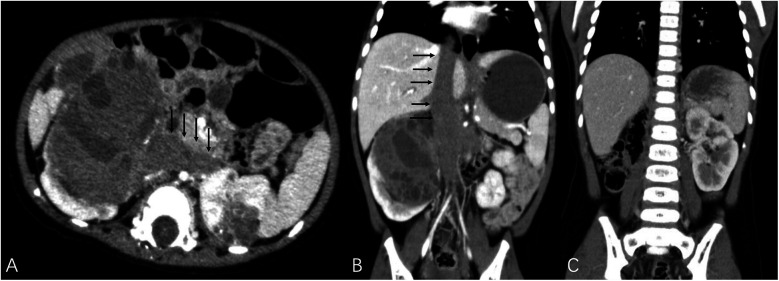
Black arrows demonstrated tumor thrombus invading bilateral renal veins and extending to the inferior vena cava as well as the atrium. **(A,B)** TUMORS – NS (both kidney units) = 16. The patient underwent right RN and left NSS with assistance of cardiopulmonary bypass. **(C)** The imaging data of postoperative reexamination.

**Figure 3:**
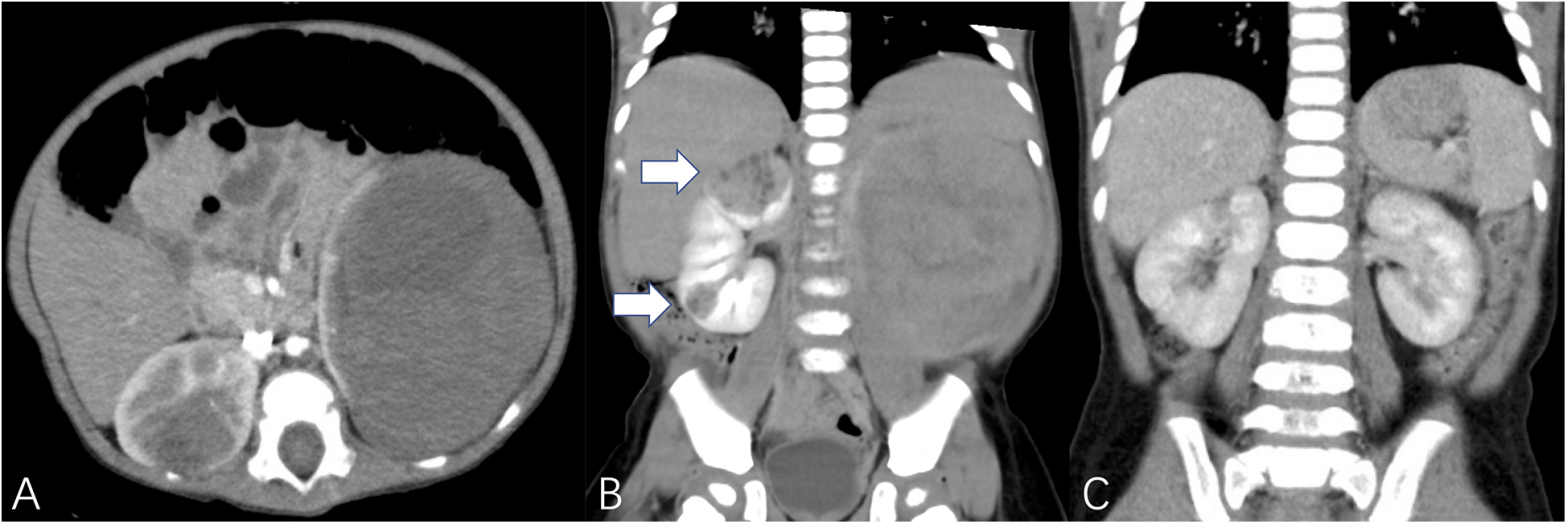
White arrows demonstrated multiple tumors. **(A,B)** TUMORS – NS (right kidney unit) = 1 + 3 + 3 + 3 + 1 + 2 = 13, TUMORS – NS (left kidney unit) = 1 + 2 + 1 + 2 + 3 + 3 = 12, patient underwent bilateral NSS. **(C)** The imaging data of postoperative reexamination.

The study included a total of 43 patients, comprising 23 females (53.5%) and 20 males (46.5%), with a median age of 17 months (range: 1–135 months). This study identified 32 cases of BWT (74.4%), and 11 cases of unilateral renal tumor (25.6%). A total of 70 kidney units were included, with 13 (18.6%) undergoing radical nephrectomy (RN) and 57 (81.4%) receiving NSS (Table 2). Within the NSS group, tumor resection *in vivo* was performed for tumors in 37 kidney units (52.9%), while tumor resection *ex vivo* via bench surgery with kidney auto-transplantation were carried out for 20 kidney units (28.6%) (Figure 1). The presence of tumor thrombus was observed in 4 units (5.7%) (Figure 2), while multiple tumors were detected in 16 units (22.9%) (Figure 3).

**Table 2:** Patients, tumor and operative data.

Parameters	Value (range or *n*%)
Patient number	43
Gender	
Female	23 (53.5%)
Male	20 (46.5%)
Age (m)	17 (1–135)
Histology	
Unilateral renal tumor	11 (25.6%)
Unilateral WT	6 (14.0%)
Renal cell carcinoma	1 (2.3%)
Cystic nephroma	1 (2.3%)
Multilocular renal cysts	1 (2.3%)
Renal angiomyolipoma	1 (2.3%)
Mesenchymal tumor	1 (2.3%)
Bilateral WT	32 (74.4%)
Synchronous bilateral WT	28 (65.1%)
Metachronous bilateral WT	4 (9.3%)
Kidney units	70^a^
Side	
Left	32 (45.7%)
Right	38 (54.3%)
RENAL system	
Low complexity group	13 (18.6%)
Moderate complexity group	16 (22.9%)
High complexity group	41 (58.6%)
TUMORS system	
Low complexity group	13 (18.6%)
Moderate complexity group	34 (48.6%)
High complexity group	23 (32.9%)
Surgical procedure	
RN	13 (18.6%)
NSS	57 (81.4%)
Tumor resection *in vivo*	37 (52.9%)
Tumor resection *ex vivo*	20 (28.6%)

^a^ The removal of 5 kidney units took place at other centers.

All kidney units were also scored according to RENAL nephrometry system, and a significant statistical correlation between the scores of the two systems was identified (*r* = 0.857, *P* < 0.01). According to the RENAL nephrometry scoring system, 13 (18.6%), 16 (22.9%), and 41 (58.6%) kidney units were categorized as having low, moderate, and high complexity respectively. Additionally, based on the TUMORS nephrometry scoring system, 13 (18.6%), 34 (48.6%), and 23 (32.9%) kidney units were classified as low, moderate, and high complexity respectively.

Table 3 revealed categorical data relationships. The findings of our study indicated that tumors exhibiting thrombus, multiple tumors, and collecting system involvement were more likely to contribute to higher complexity. The classification of tumor complexity was not dependent on whether the tumors were unilateral or bilateral. Tumors that underwent RN or *ex vivo* removal exhibited higher complexity, however, the complications of NSS were not statistically significant in relation to tumor complexity. Additionally, no statistically significant difference was observed for positive margins.

**Table 3:** Comparison of tumor complexity according to TUMORS nephrometry.

	Low complexity	Moderate complexity	High complexity	*P*-value
Thrombus				0.001
Present	0	0	4	
Absent	13	34	19	
Urinary collecting system				<0.001
Not involved (*n* = 47, 67.1%)	13	26	8	
Involved (*n* = 23, 32.9%)	0	8	15	
Number of tumors				0.035
Single tumor	12	28	13	
≥ 2 tumors	1	6	10	
WT types				0.468
Unilateral renal tumor	1	8	2	
Bilateral WT	12	26	21	
Surgical approach				0.002
RN (*n* = 13, 18.6%)	0	5	8	
NSS (*n* = 57, 81.4%)	13	29	15	
NSS modality				<0.001
*In vivo* (*n* = 37, 52.9%)	13	20	4	
*Ex vivo* (*n* = 20, 28.6%)	0	9	11	
Surgical margin of NSS				0.104
Positive (*n* = 3, 5.3%)	0	1	2	
Negative (*n* = 54, 94.7%)	13	28	13	
Complications of NSS*				0.180
Present (*n* = 17, 29.8%)	0	11	6	
Absent (*n* = 40, 70.2%)	13	18	9	

*Complications included urinary fistula, active bleeding, and tumor recurrence, thrombosis.

## Discussion

The use of NSS in pediatric renal tumors has garnered attention. Despite the absence of statistical evidence indicating disparities in short-term outcomes between NSS and RN in pediatric patients (9), considering superior long-term renal function and overall comparable results, NSS may be deemed more favorable (10, 11). The advancement of surgical techniques has facilitated the successful performance of NSS, even in cases involving renal hilum (12, 13). The selection of appropriate candidates for NSS necessitates a thorough preoperative imaging assessment. Pediatric renal tumors differ from those in adults, and the decision to perform NSS should base on individualized considerations for children. We have developed a TUMORS nephrometry scoring system, where “TUMORS” represents the acronym for thrombus, urinary collecting system, multifocality, outward property, radius, and site; this acronym is comprehensible and easy to memorize.

In this system, the tumor thrombus serves as the first assessment indicator. Renal tumor with thrombus not only indicates a high level of aggressiveness but also objectively increases the surgical difficulty. Theoretically, the presence of tumor thrombus in renal tumors is technically challenging and excludes the possibility of NSS, and the absence of thrombus is one of the prerequisites for performing NSS in unilateral WT. Therefore, we directly defined tumor with thrombus as high complexity. Previous studies have demonstrated that in cases of T3 renal cell carcinoma complicated with tumor thrombus, if the tumor is confined solely to the renal vein, there still exists a possibility for NSS to be performed (14–17). A patient in our cohort, who had BWT and right renal venous thrombus extending to the left renal vein, superior and inferior phrenic vena cava, as well as the atrium, underwent right RN and left NSS with cardiopulmonary bypass support, and postoperative follow-up demonstrated favorable renal function. It has been reported that NSS is a feasible and safe option for patients with BWT or horseshoe kidney when the branch or accessory renal veins were present (18). The existence of these rare yet successful cases demonstrates the feasibility of implementing NSS for kidney tumors with thrombus when managed by a proficient medical team, especially in cases involving only thrombus within a segmental renal vein. It is worth noting that pediatric patients exhibit particularly thinner vascular channels compared to adults. In our cohort, NSS was conducted on patients presenting with tumor thrombus in the renal vein; however, postoperative vascular embolism was still observed. This may be attributed to the invasion of the vascular wall by thrombus. Consequently, we assigned a value of 16 points to cases involving tumor thrombus, regardless of mayo thrombus grade.

The second indicator is renal collection system. Most renal tumors originate in the parenchyma and involve the urinary collecting system at an advanced stage. Only a few cases have been reported where the tumor solely occupied the urinary collecting system without involvement of the renal parenchyma (19). The presence of hematuria is frequently observed in cases where tumors infiltrate the urinary collecting system. This indicator is scored based on analyzing imaging data and assessing the presence or absence of hematuria, facilitating a more intuitive evaluation of the correlation between the tumor and the urinary collecting system. Our TUMORS system integrates tumor-related factors and objective image-based factors, thereby reducing inter-observer variability.

The third evaluation indicator is based on the number of tumors, which provides a valuable supplement to the RENAL nephrometry scoring system by addressing the assessment of multiple tumors. In our cohort, the complexity of tumors showed no statistical difference between patients with unilateral renal tumors and those with BWT. Although BWT has been reported to exhibit a higher susceptibility to the development of multiple tumors, treatment for multiple adjacent tumors may involve considering them as a single but enlarged tumor to facilitate a partial nephrectomy procedure. The surgical experience we have gained previously has demonstrated that the size of the tumor does not significantly impact surgical planning (12).

The designations (O), (R), and (S) in the TUMORS nephrometry scoring system correspond to (E), (R), and (L) in the RENAL nephrometry scoring system respectively. The anterior/posterior indicator is excluded in our TUMORS system, despite reports suggesting that retroperitoneal resection of BWT may reduce bowel damage and decrease the occurrence of urinary fistula (20). The transperitoneal route remains the predominant approach for the surgical treatment of pediatric renal tumors. Therefore, the anterior or posterior of the tumor within the kidney does not significantly affect the surgical protocol (21). The diameter of WT is always larger than that of RCC, as confirmed in our previous study (22). The utilization of 4 cm and 7 cm as indicators stems from their established maturity in assessing RCC stage and guiding surgical treatment, hence our adoption. The determination of a more appropriate cut-off value to distinguish tumor diameter in children requires further investigation.

The complexity of each kidney unit in our cohort was assessed according to the RENAL nephrometry and TUMORS nephrometry scoring systems, and a statistically significant correlation between the two systems objectively validates the feasibility of our designed TUMORS nephrometry scoring system. Our refined scoring system did not alter the low complexity category, while more kidney units were classified as moderate complexity, aligning with our subsequent clinical practice. Based on our TUMORS system, univariate analyses revealed that thrombus, urinary collecting system invasion and multiple tumors contributed to higher complexity. The primary impact of NSS on prognosis lies in its ability to decrease the occurrence of proteinuria and hypertension (23). Whether utilizing the RENAL nephrometry system or TUMORS nephrometry system, our objective is to provide potential guidance for clinical surgical treatment. In our cohort, each kidney unit was assessed and scored individually, but 74.4% units were from bilateral WT. It would be more appropriate to conduct K-M survival analysis for patients with unilateral kidney tumor between the RENAL score high complexity group and TUMORS high complexity group. Moreover, the complications and positive margins of NSS were not statistically significant in relation to tumor complexity in our study. The implementation of our NSS, particularly the application of *ex vivo* tumor resection with kidney auto-transplantation, may be attributed to this condition. During the *ex vivo* tumor removal procedure, surgical margins can be repeatedly examined and the renal collecting system can be meticulously repaired. The results of our study revealed that the RN group exhibited higher tumor complexity, as did the *ex vivo* NSS group. Our TUMORS nephrometry scoring system proves beneficial in identifying pediatric patients who are suitable candidates for NSS.

It is important to point out that while preoperative imaging has been reported to have an 87% accuracy rate in predicting the likelihood of NSS (24), all prediction models are merely referential. The successful implementation of NSS relies more heavily on the practitioner's clinical experience and surgical expertise. Previous kidney tumor scoring systems were proposed in the era of *in vivo* tumor resection. Tumor removal *ex vivo* with kidney auto-transplantation is the ultimate approach to NSS, requiring validation through multiple kidney nephrometry systems. The subcategorization based on quantitative scores to determine surgical modalities is not currently feasible with our TUMORS nephrometry scoring system. Under the premise of guaranteeing the risks associated with surgery and anesthesia, an appropriate NSS strategy should be carefully selected for each pediatric kidney tumor with the aid of kidney nephrometry system. The proposed TUMORS nephrometry scoring system included more indicators, however, the sample in this study was limited to a single center. The validation process requires further study and a larger sample size from multiple centers.

## Conclusions

The TUMORS nephrometry system incorporates a broader range of factors to assess comprehensively the anatomical characteristics of pediatric renal tumors, thereby facilitating the identification of suitable candidates for NSS. The likelihood of tumor resection *ex vivo* and RN increases with the complexity of tumors. Nevertheless, this system still requires further validation using data from additional centers and lager sample sizes.

## Data Availability

The raw data supporting the conclusions of this article will be made available by the authors, without undue reservation.
